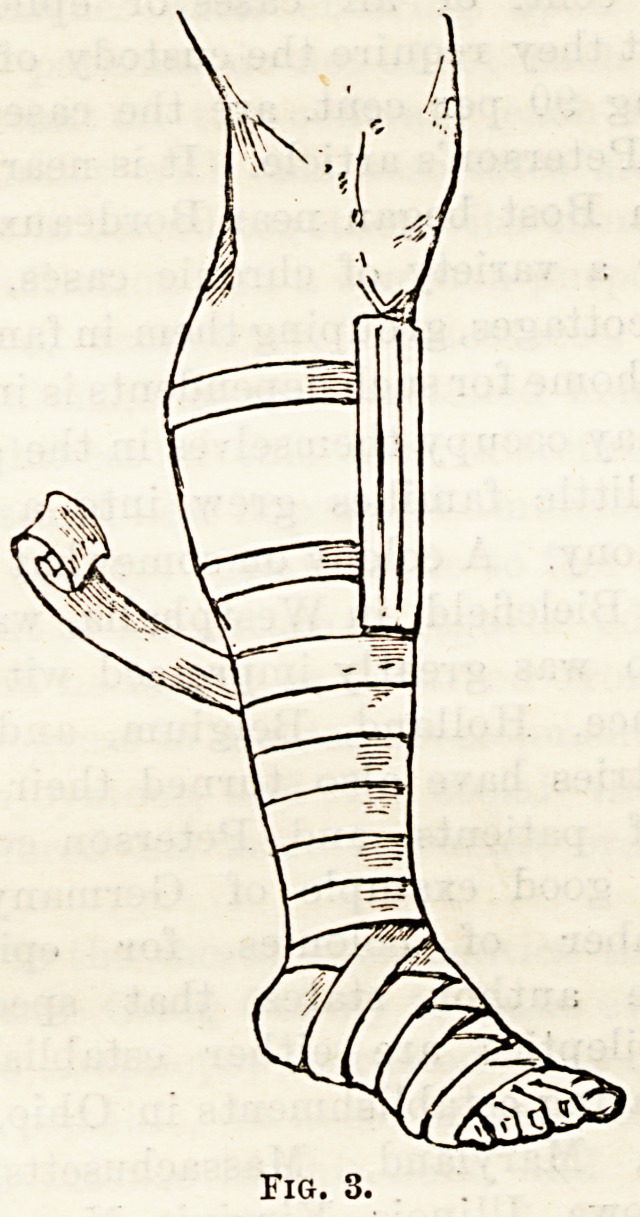# Ununited Fracture: Its Causes and Treatment

**Published:** 1897-01-02

**Authors:** E. Percy Paton

**Affiliations:** Surgical Registrar to Westminster Hospital.


### MEDICAL PROGRESS AND HOSPITAL CLINICS.

[*The Editor will be glad to receive offers of co-operation and contributions from members of the profession. All letters should be addressed to* THE EDITOR, AT THE OFFICE, 28 & 29, SOUTHAMPTON STREET, STRAND, LONDON, W.C.]

### UNUNITED FRACTURE: ITS CAUSES AND TREATMENT.

E. PERCY PATON, M.D., M.S., F.R.C.S., Surgical Registrar to Westminster Hospital.

Ununited fractures, though not at all common, considering the frequency with which fractures occur, are still sufficiently so to demand careful attention, more especially so as the more complete forms of want of firm union give rise to such serious loss of function in the affected limb. Several varieties of defective union are met with, but they may all be classed under two main heads, namely, cases in which there is merely delayed consolidation, and those which have gone on to the formation of some form of false joint. This distinction is not merely convenient for descriptive purposes, but the treatment of the two classes of cases is essentially different.

FIG. 1.

FIG. 2.

Delayed consolidation results from want of proper callus formation or imperfect ossification after its deposition; it may result in the formation of a false joint, but usually does not, and is the most usual form of mal-union. A false joint may mean practically no union at all, resulting in a "flail limb," or there may be more or less fibrous union, or a joint may be formed which is more or less like a real one, and has in some cases been found to have capsule, synovial membrane, and cartilage, as in a normal joint, and even osteoarthritic changes have in rare cases been seen in pseudo-joints (see Figs. 1 and 2). The secondary results of non-union on the limb vary according to the freedom of movement which is present, but there is usually much wasting of muscle, and so, loss of functional power; while, if it occurs in the young, a good deal of atrophy of the whole limb generally follows. The causes of want of union have been classified as general and local. Of the former, any acute or wasting disease, syphilis, scurvy, &c., have been held responsible; but it is doubtful to what extent any of these act efficiently, and they are certainly of much less moment than the local causes, such as the following: (1) Wide separation of the fragments, and consequently want of proper apposition; a common example of this occurs in the ordinary transverse fracture of the patella, or fracture of the olecranon, which always unite by fibrous tissue only; (2) interposition of some foreign body between the fragments, such as a piece of muscle or tendon, part of the capsule of a joint, or, in fracture of the lower jaw, a loosened tooth, &c.; (3) imperfect treatment, where sufficient rest is not obtained. This is well seen in cases where fractures have occurred, far from skilled advice and treatment—for example, in sailors at sea. It may, however, occur when splints have been used without properly fixing the fragments, by including the joints above and below the injury, or possibly the circulation may have been interfered with by improper bandaging, but this seems very unlikely, without producing serious damage to soft parts as well. Imperfect fixation is especially likely to occur in oblique fractures of the tibia, the obliquity not only permitting but also aiding in a sliding movement of one fragment on the other, often not easy to prevent; (4) lastly, may be grouped together such troubles as possible injury to the blood or nerve supply of the bone at the original accident, disease of the broken ends, such as necrosis, either from constitutional causes or the severity of the injury, or in a compound fracture from extensive suppuration, &c.

The diagnosis of want of union is obviously easy, but it not always so to decide if there is merely delayed consolidation or the much more troublesome condition of false joint formation. Lapse of time is one of the most important points, while the freedom of movement with smooth sliding of the bones over each other without the feeling of crepitus, may help, along with careful examination of the broken ends of the bones, for the purpose of trying to feel if any cupping or socket has been formed on one of them, such as usually occurs in a false joint.

The prognosis is very uncertain, but depends especially on the amount of mobility and of functional disturbance of the limb. Cases which are merely examples of delayed consolidation usually rapidly become firm under appropriate treatment, but a fully-formed false joint is often very difficult to treat, even by extensive operative interference.

The treatment which is applicable to any particular case depends largely upon the pathological condition present, and here it may be premised that nothing short of an open operation is of any use in cases where a false joint has formed.

When, however, a union is found to be imperfect under ordinary treatment, even after the usual time for firm consolidation is long past, the best thing then to do is to anæsthetise the patient, and with considerable violence rub the ends of the bone together with a view of setting up fresh irritation, and therefore fresh activity about the fracture. This is then put up in some fit apparatus; plaster, either in the form of a Croft splint or ordinary plaster bandages, being especially suitable, which may be left on three or four weeks before the limb is taken down for examination, when it will often be found that union is much firmer, and by continuing the splint for some time satisfactory consolidation is obtained. Should this form of treatment be found unsuccessful after careful trial, that of constriction of the limb above and below the seat of injury has been recommended, though I have no personal experience of its

FIG. 3.

efficiency. The seat of fracture is carefully protected from pressure, and so what is described as an "active congestion" of the part is maintained for some days, or even longer, and this has been found in some cases of delayed union to favour consolidation. The method used is as follows: The fracture and immediately surrounding parts are left free from pressure by arranging above and below it two narrow pads, about an inch wide, upon which rest the upper and lower ends of a piece of kettle-holder splinting. (See Fig. 3.) The limb is then bandaged from its extremity upwards over the splint, and to same distance above it; the bandage, which must be laid very evenly, is applied with considerable firmness, the result being that a certain amount of congestion is produced at the fracture. This is kept up for some days, or longer, the condition of the limb being carefully watched. Subsequently a fixed apparatus is again applied.

The success of this method appears to depend on the careful attention to an exact regulation of the pressure applied by the bandages, so that the firmest possible pressure may be applied below the fracture consistent with the safety of the soft parts; while above, it shall be firm enough only to influence the venous circulation to some extent, and prevent the too rapid return of blood. Owing to the risk of applying the bandages too tight, it has been advised to put them on tightly for, say, six hours twice daily, and allow intervals for rest between. Another good method in obstinate cases is to put on as fixed an apparatus as possible, such as plaster or a Thomas' hip or knee splint, and let the patient get up and about, when it is sometimes found that, with the improved general condition and the irritation of slight movement, consolidation will take place. Should this treatment fail, various other methods have been advised short of actual open operation, such as prolonged extension of the limb, irritation of the intermediate substance which lies between the fragments by injection of irritating fluids, acupuncture, galvano-puncture, and use of a seton, &c.; but none of these appear to be very effectual, and some of them are by no means free from serious danger much greater than that of an open operation.

Should the measures which have been described above be unsuccessful, or should a false joint have formed, some form of open operation will be necessary, and one of the following procedures will have to be done. First, aseptic ivory or bone pegs, or even metal pins, may be driven into the fragments just above and below the fracture, after having exposed them by incision. This measure, in cases of delayed consolidation, will often cause sufficient irritation to produce firm union. It is, however, useless in cases of false joint. In these latter cases a much more extensive exposure of the mal-union is necessary, so that the ends of the bones may be re-resected, the false joint being removed either completely or partially, but in any case sufficiently to permit freshened ends of bone to come together, which may then be pegged into their new fixed position by means of ivory or bone pegs. Aseptic steel screws have been recommended for this purpose, but in my experience they do not seem to have the advantages which they might seem to have, apparently for two reasons, namely, that the screws are put in obliquely through the bone ends, and also that not infrequently the bone ends are somewhat atrophied and soft, and so the screws do not "bite" well, and tend, therefore, to act merely as pegs. Whatever form, however, of fixation method may be used, two things are of primary importance, namely, in the resection to preserve the periosteum with the greatest care, and to get firm fixation afterwards, both by means of efficient pegging and also by the splints used subsequently.

The difficulty, however, of getting satisfactory union after a false joint has formed is much increased by the fact that after resection of the joint the interval thus formed may be so great that it is not possible to get the bones into apposition again, and attempts have been made, not always very successfully, to fill in the gap by means of bone transplantation. Four methods have been used, which may be shortly indicated as follows: Either a piece of bone may be separated from the upper or lower fragment, and turned round so as to more or less fill up the gap, leaving it attached to the fragment from which it came by means of the periosteum; or merely a piece of periosteum may be transplanted and turned into the gap; or a piece of bone may be removed from one or other fragment and fixed in the gap after all its connections with its source have been severed; or a piece of bone from another animal, such as a rabbit or dog, may be used in the same manner. Of these methods the first seems the most satisfactory; but none of them are very successful, and in all of them to obtain the greatest measure of success the strictest asepsis must be maintained. Should a careful trial of several of the above-mentioned forms of treatment fail in any particular case, as they sometimes will, two other courses are still open, namely, either to palliate the deformity as far as possible by some orthopædic apparatus, or, if the limb be merely a troublesome appendage in its damaged condition, it may be necessary to remove it by amputation.

## Figures and Tables

**Fig. 1. f1:**
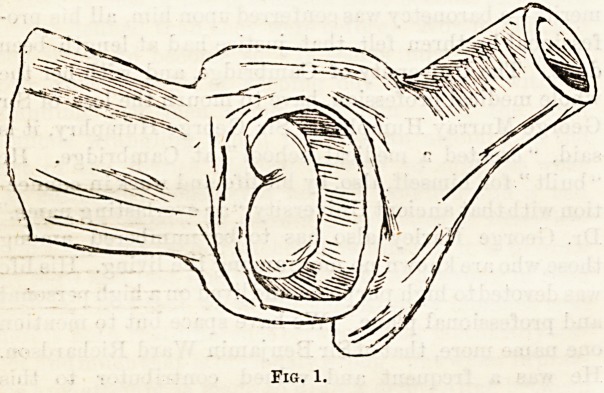


**Fig. 2. f2:**
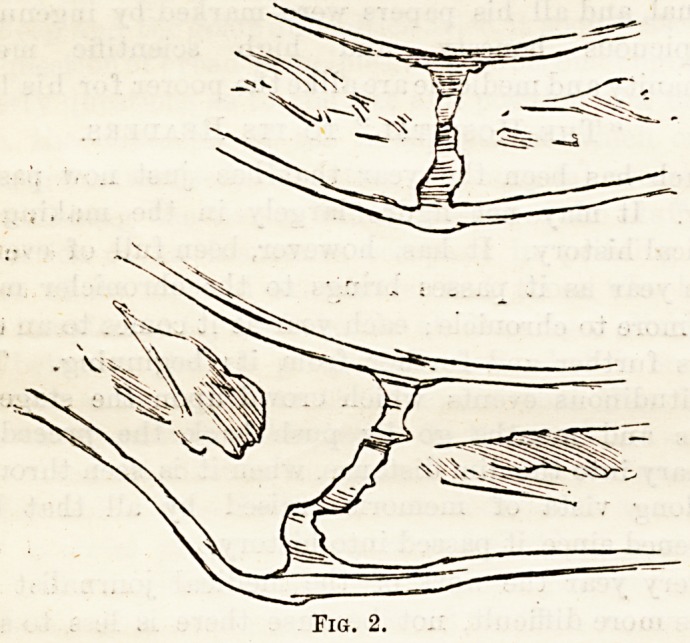


**Fig. 3. f3:**